# Lost His Appetite

**Published:** 1915-05

**Authors:** 


### Lost His Appetite.

One has often to laugh at the cute sayings of children, and it is a pleasure to me to send you something original. I know this is original, because Mrs. D. was with a party of ladies when it happened.

A lady with her little boy was attending a sewing circle and to keep the little boy quiet his mother had bought him several nice apples. During the afternoon he ate three or four of the apples and then came to his mother with the statement that he had lost something.

"Well, what was it, my son?"

"I lost my appletite."

When I came into the room the mother told him to "Tell the gentleman what you lost. He straightened up to his full height and, raising his head, with pleasure just bulging out of his eyes, he in a loud voice exclaimed:

"I lost my appletite."

I thought at once of your funny page—and so here it is.—Dr. D., in *Drug News.*

